# Biocompatibility Study of Hydrogel Biopolymer Scaffold with Encapsulated Mesenchymal Stem Cells

**DOI:** 10.3390/polym15061337

**Published:** 2023-03-07

**Authors:** Marfa N. Egorikhina, Lidia B. Timofeeva, Daria D. Linkova, Yulia P. Rubtsova, Marina L. Bugrova, Irina N. Charykova, Maxim G. Ryabkov, Irina I. Kobyakova, Ekaterina A. Farafontova, Diana Y. Aleynik

**Affiliations:** Federal State Budgetary Educational Institution of Higher Education, Privolzhsky Research Medical University of the Ministry of Health of the Russian Federation, 603005 Nizhny Novgorod, Russia

**Keywords:** scaffold, mesenchymal stem cells, tissue-engineered constructs, preclinical studies, biocompatibility, regeneration, biopolymers

## Abstract

One of the key and actively developing areas of regenerative medicine is tissue-engineering. There is no doubt that the use of tissue-engineering products can have a significant impact on the efficiency of repair of damaged tissues and organs. However, before being used in clinical practice, tissue-engineering products require thorough preclinical studies to confirm their safety and efficacy, both with in vitro models and in experimental animals. This paper presents preclinical studies of a tissue-engineered construct, based on a hydrogel biopolymer scaffold carrier (consisting of blood plasma cryoprecipitate and collagen) with encapsulated mesenchymal stem cells, to evaluate its biocompatibility in vivo. The results were analyzed using histomorphology and transmission electron microscopy. It was shown that when implanted into animal (rat) tissues, the implants were completely replaced by connective tissue components. We also confirmed that no acute inflammation occurred in response to the scaffold implantation. The observed processes of cell recruitment to the scaffold from the surrounding tissues, the active formation of collagen fibers and the absence of acute inflammation testified that the regeneration process was ongoing in the implantation area. Thus, the presented tissue-engineered construct shows promise for becoming an effective tool for regenerative medicine in the future and may be used, in particular, to repair soft tissues.

## 1. Introduction

Tissue-engineering is the most interesting and promising area of regenerative medicine. Today, the development of tissue-engineering is aimed at developing artificial tissue constructs (TECs—tissue-engineered constructs) for the regeneration of damaged organs and tissues [1]. However, the successful use of tissue-engineered products requires an understanding of the product’s behavior in a living organism, in particular the features of its biodegradation and interactions with surrounding tissues, along with the transformations undergone during the regenerative process. All this allows prediction at the preclinical in vivo research stage of the efficacy and safety of the TEC being developed.

Some of the key studies recommended by ISO 10993-6-2021 [2] at initial preclinical studies of medical devices are in vivo biocompatibility investigations. The term biocompatibility of materials is most commonly understood as the compatibility of a particular material with a biological system. However, despite this term being widely used in the scientific community, the specific mechanisms and phenomena making up such biocompatibility are not clearly defined. Biocompatibility testing evaluates the medical device safety in terms of the physiological effects it causes [3]. Biocompatibility studies are also applicable to TECs, since the scaffold carriers or cell matrices included in the constructs are essentially medical devices.

The ISO 10993-6-2021 recommendations on cell carriers state the following: “Materials used as matrices (scaffolds) for tissue-engineered medical products should not be investigated as part of a finished medical device with pre-introduced cells and/or proteins, since the animal immune response to the cell/protein components of these products and the response of the cells themselves when injected into the animal may interfere with the final local tissue response, complicating its interpretation”. At the same time, it should be noted that it is not always correct to evaluate the biodegradation and biotransformation of a cell carrier without cells, since cell-containing and cell-free products may behave differently within a biological system. When studying a tissue-engineered product in vitro, we are dealing with a “closed” system consisting of two main components: cells and their carrier. At the same time, a dynamic interaction exists between the components of this system: when the carrier influences cell behavior through external signals, and when the cells, at the same time, transform their microenvironment, maintaining its homeostasis [4,5,6,7]. When switching to in vivo studies, it should be taken into account that the object of study, the living organism, is an open system, and dynamic interaction will be observed between the organism, the tissue-engineered product and its components (organism−whole TEC, organism−TEC matrix/scaffold, organism−TEC cell component, cells−matrix/scaffold). Thus, it is important to discover how the product interacts with the organism, what transformations it undergoes and how it affects the organism. Therefore, it is advisable to investigate both the biocompatibility of the matrix when separate from the cellular component and the biocompatibility of the tissue-engineered product as a whole.

At the same time, when evaluating the biocompatibility of a tissue-engineered product populated with cells, a number of questions about immunocompatibility may arise at the stage of product research in animal models. It is well known that tissue-engineered products are not inert, but have high biological activity, and the presence of this invariably leads to the need to consider the histocompatibility and immunogenicity of such products [8,9]. For example, tissue-engineered products intended for application in humans use human cells. However, when bioproducts with human cells are implanted into animal tissues, the tissue-engineered products pass into xenogeneic conditions, and this a priori increases the risk of the recipient’s body developing a conflict with the implanted construct [10,11,12]. Moreover, the biomaterial implantation process may provoke a foreign body reaction (FBR)—a nonspecific immune response of the body that may significantly worsen implant engraftment [13]. All of this may end up causing false negatives. In this regard, in preclinical studies aimed at proving the safety and efficacy of TECs, the products being developed may be modified towards a “homologous drug” strategy. For this reason, the development of homologous TEC models for certain species of laboratory animals is highly relevant [8,13].

It should be added that the recommendations of ISO 10993-6-2021 include some requirements for control specimens: “The local tissue response to the test specimen shall be evaluated in comparison with the response to a control specimen made of similar materials with established clinical applicability and biocompatibility”. This condition somewhat complicates TEC investigations, since finding very close analogs approved for medical use is often quite a big problem. This happens because most TECs are original developments and may, in practice, have no analogs; in addition, though original laboratory developments are very numerous, only a small proportion is represented on the international market [14], and this also complicates the search for analogs.

Equally important for the correct evaluation of a tissue-engineered product is to understand how its biodegradation or biotransformation occurs in vivo. In recent years, many developments in the field of tissue-engineering have focused on the concept of bioresorbable scaffolds. Scaffolds made of bioresorbable materials, in addition to their physical support function, allow the damaged tissue to restore its natural anatomical configuration without causing chronic inflammation from constant contact with the foreign body [15,16,17,18,19,20]. The lack of need for repeated surgical interventions to remove such implants is also an undeniable advantage of bioresorbable materials [21]. However, the disadvantages of these materials may be in their having insufficient mechanical strength, and decomposition rates that are inappropriately low or too high [15,22]. Therefore, when developing biodegradable scaffolds, a balance should be observed between the mechanical strength of the implant and maintaining a certain biodegradation rate after implantation [21,22]. In this case, only in vivo studies in animal models are able to provide for appropriate evaluation of scaffold and TEC biotransformation when implanted into a living organism.

The aim of our study was, comparatively, to evaluate in vivo the biocompatibility of an original biopolymer hydrogel scaffold carrier and of tissue-engineered constructs, based on this scaffold carrier, but with encapsulated mesenchymal stem cells.

## 2. Materials and Methods

The study protocol was approved by the local ethical committee of the FSBEI HE PRMU MOH (Nizhny Novgorod, Russia) (approved by the local ethics committee on 10 March 2021, protocol No. 5). All research protocols complied with international ethical requirements, including the European Convention for the Protection of Vertebrate Animals used for Experimental and Other Scientific Purposes (1986, 1998).

### 2.1. Rat Mesenchymal Stem Cell (MSC) Culture

For the animal studies (in rats), MSC cultures were obtained from the subcutaneous adipose tissue of an intact animal. Cells were isolated using mechanical disaggregation and thermal enzymatic treatment with a collagenase solution (Sigma-Aldrich, Darmstadt, Germany), followed by filtration and cultivation in a complete growth medium under CO_2_ incubator conditions (5% CO_2_, +37 °С, absolute humidity). The complete growth medium consisted of DMEM F-12 (Gibco™, Thermo Fisher, Waltham, MA, USA), 10% fetal bovine serum (FBS) (Gibco™, Thermo Fisher, Waltham, MA, USA), 2% glutamine, and antibiotics (penicillin/streptomycin) (LLC PanEco, Moscow, Russia). A 0.25% trypsin solution in Versen (Gibco™, Thermo Fisher, Waltham, MA, USA) was used for the passage. When a subconfluent monolayer (up to 80%) was reached, the culture was transplanted. Cultures of 3–4 passages were used in the study.

The rat MSCs were identified with a FACS Canto II flow cytometer (Becton-Dickison, East Rutherford, NJ, USA) using specific monoclonal antibodies Anti-Mo/Rat CD 90.1 (Thy1.1) PE clone: HIS51 and Anti-Rat CD 45 FITC clone: OX1 (eBioscience) with appropriate isotypic controls. According to the results of phenotyping, more than 90% of the cells had typical MSC characteristics: CD90+, CD 45−.

Cell viability was 98–99% before introducing them into the composite to produce the cell-containing scaffolds and the cell-based collagen implants.

### 2.2. Scaffold Forming

To form cell-free scaffolds (CFSs) and cell-containing scaffolds (CCSs), a composite based on human blood plasma cryoprecipitate was used [23]. To PEGylate the cryoprecipitate component, PEG-NHS (Sigma-Aldrich, Germany) was injected. A 2% collagen solution (marine collagen isolated from cod skins) was added to the PEGylated cryoprecipitate, pH = 7.2 to 7.4 [24]. In those studies, during CCS formation, an MSC in PBS suspension was introduced into the composite. As the CCS was intended for future use in humans, human adipose tissue MSCs were used. In our study of CCSs, the human MSCs were substituted with rat adipose tissue MSCs according to the “homologous drug” strategy. The rat cell concentration was 1.2 × 10^5^ per 1 mL of composite. During the formation of the cell-free scaffolds, a phosphate buffer solution (PBS) was injected into the composite in a 7:1 ratio. To polymerize the composite, a thrombin-calcium mixture was added: 80 IU/mL thrombin (Sigma-Aldrich, Germany) in 1% CaCl_2_ solution. The CCSs were cultured for 3 days in a complete growth medium under CO_2_ incubator conditions.

**Figure 1:**
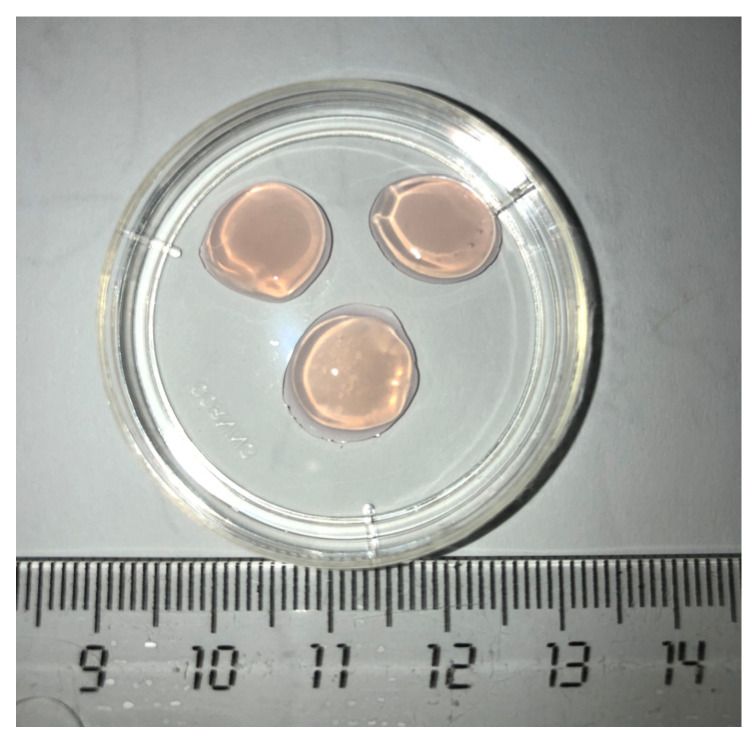
Appearance of the studied biopolymer scaffold.

Before the experiment, the CCSs and CFSs were washed three times with the phosphate buffer solution and then sliced using a template into equal 1 cm^2^ fragments. A macroscopic image of the scaffold samples is shown in Figure 1.

### 2.3. Formation of Cell-Containing and Cell-Free Control Implant Specimens

A collagen-based biopolymer porous matrix (a clinically approved commercial product, COLLATAMP^®^EG, Syntacoll GmbH, Saal an der Donau, Germany) was used as the control specimen. First, 1 cm^2^ specimens, 2 mm thick were sliced from a collagen matrix plate using a template. The cell-free specimens were introduced into the experiment as these 1 cm^2^ fragments. The following manipulations were used to obtain specimens populated with cells. Cell suspension (rat adipose tissue MSCs) was injected into each collagen matrix fragment using an insulin syringe. The concentration of cells per specimen was 250,000 units. For this purpose, rat MSC cells of the given concentration were diluted in 100 µL of phosphate buffer solution, transferred into an insulin syringe, and injected into the specimen gently, slowly, in a forward motion. Specimens of the cell-containing collagen matrix were placed individually into the wells of a 24-well flatbed. Next, 1 mL of DMEM F-12 nutrient medium (Gibco™, Thermo Fisher, Waltham, MA, USA) with 15% serum (FBS) (Gibco™, Thermo Fisher, Waltham, MA, USA) was added to each well. The flatbed with the specimens was then kept under standard СО_2_ incubator conditions. After 3 days, the specimens in the flatbed were washed three times with a phosphate buffer solution ready to be used for the experimental study.

### 2.4. Evaluation of Scaffold Biocompatibility In Vivo

The study was conducted in accordance with the recommendations of ISO 10993-6:2016 [2].

#### 2.4.1. Animal Experimental Studies

The experimental study was conducted on 60 animals (male outbred non-linear Wilson breed Wistar runoff rats weighing 200 to 250 g), using 5 animals for each of the four group types for 3 different control periods (7, 14 and 21 days). A 2 cm full depth skin incision was made in each rat on the back, to the right side of the spine. The skin was separated from the fascia to form a pocket (inward from the right side, directed from the animal’s spine to the abdomen). One of the four specimens of the material was implanted into the cavity thus formed. All samples had an area of 1 cm^2^ and a thickness of 2 mm.

Group #1 rats were implanted with a cell-free collagen matrix (Control #1; 15 rats).

Group #2 rats were implanted with a cell-containing collagen matrix with rat MSCs (Control #2; 15 rats).

Group #3 rats were implanted with a cell-free scaffold (Experiment #1, 15 rats).

Group #4 rats were implanted with a cell-containing scaffold (Experiment #2, 15 rats).

The animals were removed from the experiment as the test periods expired on days 7, 14, and 21. The following were evaluated at removal: the general condition of the animals, the appearance of the sutures and surrounding tissues, tissue color, signs of inflammation of the implant area, together with the size of the remaining implant fragment. The biomaterial sampling procedure stages were photodocumented. During surgery, the material implantation area was excised, including the capture of a muscle fragment for morphological analysis.

For all manipulations, general anesthesia was used with the animals. Overall, 60 mg/kg of Zoletil 100 (VirbacSanteAnimale, Carros, France) and 6 mg/kg of XylaVet (Pharmagist Ltd., Szeged, Hungary) were administered as intramuscular injections to achieve this.

#### 2.4.2. Morphological Analyses of the Biomaterials

The specimens obtained according to Section 2.4.1. were fixed in a 10% neutral formalin solution, then dehydrated in an alcohol battery and embedded in paraffin. Then, 5 µm thick slices were cut from the paraffin blocks using a Leica SM 2000R microtome. The slices were deparaffinized, stained with hematoxylin–eosin, dehydrated and embedded under coverslips in BioMount synthetic medium.

A NikonEclipse 80i microscope with a NikonDs-Fi1 camera was used to run morphometric processing and to create a photo archive of the resulting materials. The Nis-ElementsBR program was used for the morphometric processing of the materials. The local biological post-implantation effect was evaluated according to ISO 10993-6-2011 recommendations. A semi-quantitative evaluation system was used for each investigated biological characteristic.

### 2.5. Biotransformation Analysis of Cell-Containing Scaffolds In Vivo

#### 2.5.1. Animal Experimental Study

The study was conducted on 12 animals (male outbred non-linear Wilson breed Wistar runoff rats weighing 200 to 250 g), using 2 animals in each of the two groups (Group #1 with collagen sponge populated with mesenchymal stem cells—Control; Group #2 with cell-containing scaffold—Experiment) for each test period (3, 7 and 14 days).

All animal manipulations and the experiment were conducted in the same way as described in Section 2.4.1. As the control periods expired, animals were removed and material was taken for examination using transmission electron microscopy.

#### 2.5.2. Transmission Electron Microscopy

The specimens were prepared and examined according to standard methods [4]. The specimens were dehydrated in ascending alcohols (50 to 100%) and acetone (100%), then incubated in a mixture of 50% priming medium and 50% acetone followed by incubation in Araldite–Epon mixture. The specimens placed in the mixture were incubated for 24 h at 37 °C, then polymerized at 60 °C. Ultra-thin 75–80 nm thick slices were obtained using a Leica UC7 ultramicrotome and viewed using a transmission electron microscope (Morgagni 268D; FEI). Two TEC analyses were run during the study:

- Counting the number of cells within a 38 × 38 µm field of view of the specimen (n = 25 from each specimen);
- Analysis of structural changes in the implant itself without cells: microphotographs obtained at 14,000× magnification (n = 20 from each specimen) were processed using the ImageJ software package (National Institutes of Health). This software package calculates the percentage ratio of the examined area to the entire image area.

### 2.6. Statistical Analysis

The results were processed using nonparametric statistics involving the Mann–Whitney U-test (STATISTICA 6.0 software package).

## 3. Results and Discussion

### 3.1. Macroscopic Observations

**Figure 2:**
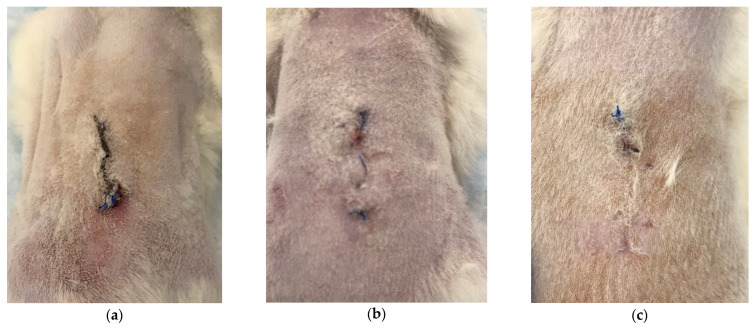
Postoperative suture appearance on day 7 (**a**), day 14 (**b**), day 21 (**c**)—representative photographs.

When the animals were removed from the experiment, their general condition was evaluated as satisfactory: the animals were active, with good appetite, and no inflammation of the surgical sutures or surrounding tissues was observed. The suture was visualized as a thin connective tissue scar (by 7 to 14 days), or a barely visible scar-like formation (by days 14 to 21) (Figure 2). The state of the postoperative suture in the control period was identical in parallel groups.

**Figure 3:**
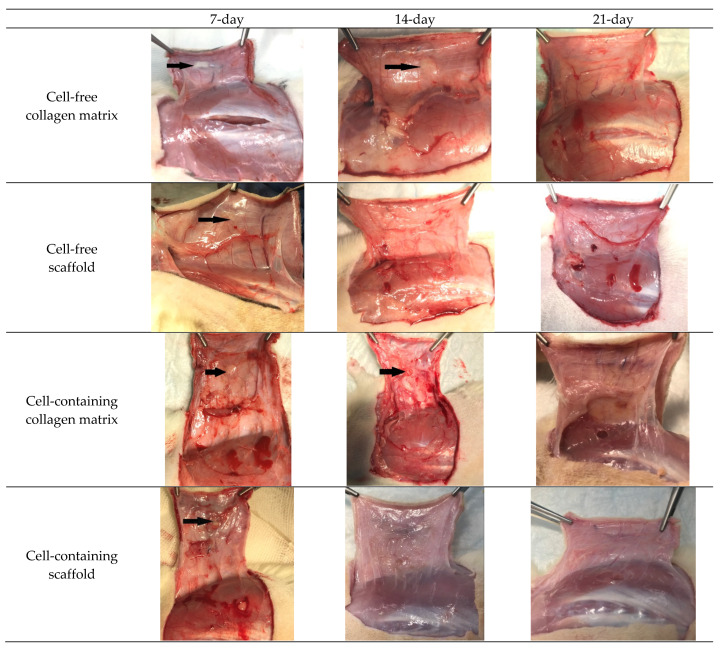
Appearance of the implantation area when material was taken. Note: The black arrows indicate implant fragments visible to the naked eye in the animal tissue structure.

When the material was taken, no inflammation was present in the area of the implantation zone, the mucosa was pink and shiny, and the blood vessels were clearly visible. As a rule, implants or the presence of fragments of them could be observed for the shorter test periods; implants could not be visualized after the longer test periods (Figure 3).

### 3.2. Results of Morphological Analyses of Cell-Free Implants

**Figure 4:**
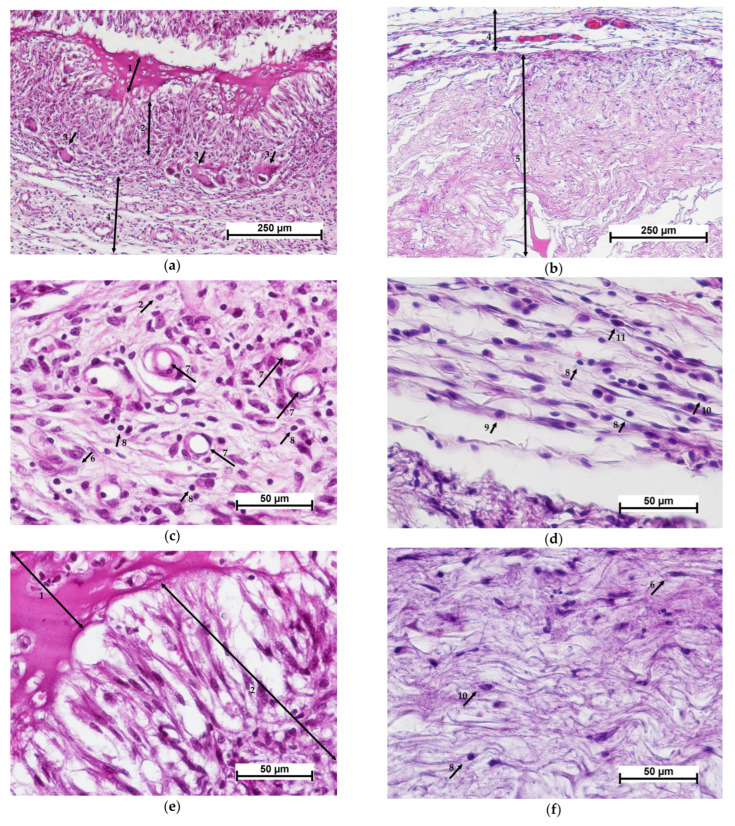
Histological picture—day 7. Comparison of cell-free scaffold (**a**,**c**,**e**) and collagen matrix (**b**,**d**,**f**). (**a**,**b**)—implant with surrounding tissue, general view, (**c**,**d**)—connective tissue structure surrounding the implant, (**e**)—CFSs and resorbing cells, (**f**)—collagen matrix structure. Staining with hematoxylin–eosin. Note: (1)—scaffold; (2)—resorbing cell (macrophage); (3)—giant multinucleated cells; (4)—surrounding connective tissue; (5)—collagen matrix; (6)—active fibroblasts; (7)—blood vessels; (8)—lymphocyte; (9)—monocyte; (10)—granulocyte; (11)—mast cell.

Morphological analysis revealed that active material resorption could be observed 7 days after cell-free scaffold implantation. The implanted matrix was fragmented and surrounded by macrophages and giant multinucleated foreign body cells (Figure 4a). Around the cluster of resorbing cells, new connective tissue was formed with active fibroblasts and macrophages, blood vessels, moderate lymphocytic infiltration and single granulocytes (Figure 4c). The scaffold was filled with cells showing signs of degenerative changes (Figure 4e). Active biodegradation of the implant was observed.

Biodegradation of the control material, the collagen matrix, was much slower. Thus, on day 7 after implantation, the control material, unlike the experimental one, retained its integrity and had a clear boundary with the surrounding connective tissue (Figure 4b,d,f). The initial stages of matrix colonization by cells could be observed, and the accumulation of cells was most evident at the border between the matrix and tissues. The surrounding tissue showed a weakly evident leukocytic infiltration, similar to the experimental specimens of the cell-free scaffold, with a predominance of mononuclear leukocytes (lymphocytes and monocytes) and macrophages, single segmented-nuclear leukocytes and mast cells. However, active collagenogenesis and neoangiogenesis were not detected (Figure 4d). Fibroblastic cells, lymphocytes, macrophages and single eosinophils could be determined in the implant structure. Some of the cells showed degenerative changes (Figure 4f).

**Figure 5:**
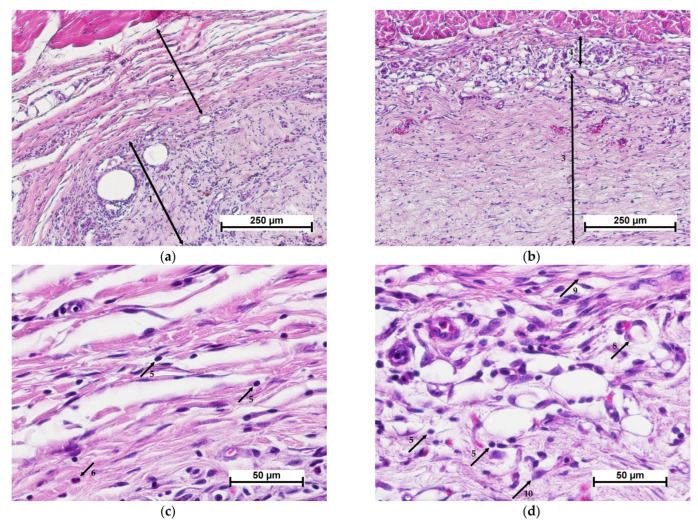

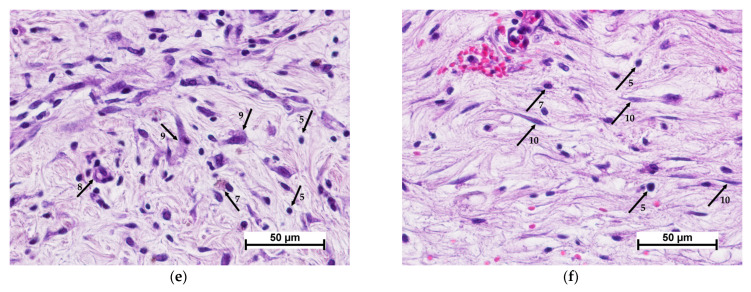
Histological picture—day 14. Comparison of cell-free scaffold (**a**,**c**,**e**) and collagen matrix (**b**,**d**,**f**). (**a**,**b**)—implant with surrounding tissue, general view, (**c**,**d**)—connective tissue structure surrounding the implant, (**e**)—connective tissue at the cell-free scaffold site, (**f**)—collagen matrix structure. Staining with hematoxylin–eosin. Note: (1)—connective tissue replacing the scaffold; (2)—connective tissue surrounding the scaffold; (3)—collagen matrix; (4)—connective tissue structure around the implant; (5)—lymphocyte; (6)—granulocyte; (7)—macrophage; (8)—blood vessel; (9)—fibroblast; (10)—fibrocyte.

Fourteen days after cell-free scaffold implantation, new connective tissue formed at the implant site could be detected, surrounded by a layer of mature connective tissue, indicating complete resorption (Figure 5a). The surrounding mature connective tissue showed edema, mild lymphocytic infiltration and single granulocytes (Figure 5c). The implanted material had been replaced by newly formed connective tissue dominated by active fibroblasts and macrophages; slight lymphocytic infiltration and foci of neovascularization were also observed (Figure 5e).

In the control specimens, unlike the experimental ones, the implanted material could still be detected on day 14, but the boundary between the implant and the surrounding connective tissue was not clearly defined (Figure 5b). Vascularization of the implanted material was observed along with its uniform colonization by cells of predominantly fibroblastic and lymphohistiocytic origin (macrophages, lymphocytes) (Figure 5f). The predominant cells of fibroblastic origin were fibrocytes without high synthetic activity. Thus, the processes of matrix resorption and its replacement by connective tissue in the control specimens had proceeded simultaneously. In the experimental specimens of the cell-free scaffold, the sequence of resorption and collagenogenesis was evident.

**Figure 6:**
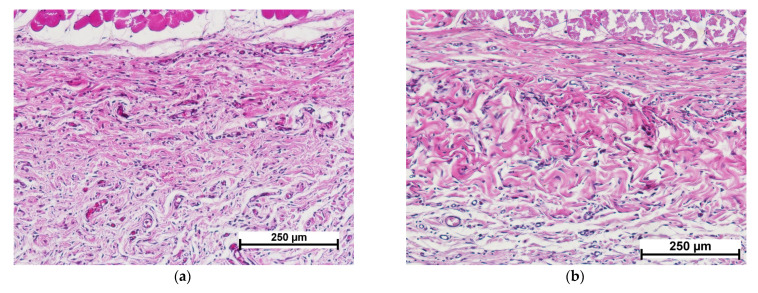

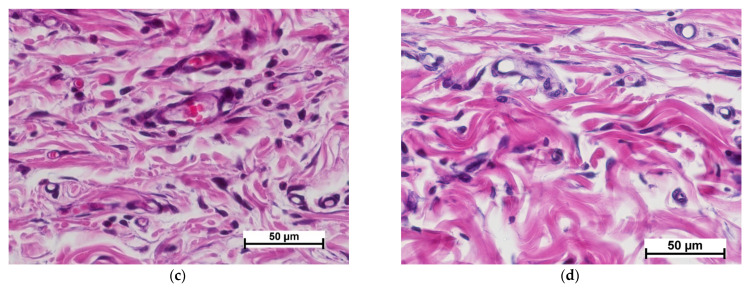
Histological picture—day 21. Comparison of cell-free scaffold (**a**,**c**) and collagen matrix (**b**,**d**). (**a**,**b**)—implant site with surrounding tissue, general view, (**c**,**d**)—connective tissue structure at the implant site. Staining with hematoxylin–eosin.

On day 21, a dense fibrous unstructured connective tissue was observed at the cell-free scaffold site (Figure 6a). The number of active fibroblasts had decreased in comparison with the previous period (day 14), and fibrocytes prevailed. Mild lymphocytic infiltration was maintained, and single granulocytes were observed (Figure 6c). Full-blooded vessels characterized the histological picture.

At the collagen matrix implantation site, as in the case of the cell-free scaffold, there was a layer of dense irregular connective tissue with a small number of blood vessels filled with blood (Figure 6b,d).

Thus, it can be concluded that neither the control nor the cell-free materials under study caused acute inflammation of the surrounding tissues. Biodegradation of the experimental material (CFS) was observed to have occurred within shorter periods compared to the control specimens. However, connective tissue had completely replaced both specimens by day 21.

### 3.3. Morphological Analysis of Results for Implants Containing MSCs

**Figure 7:**
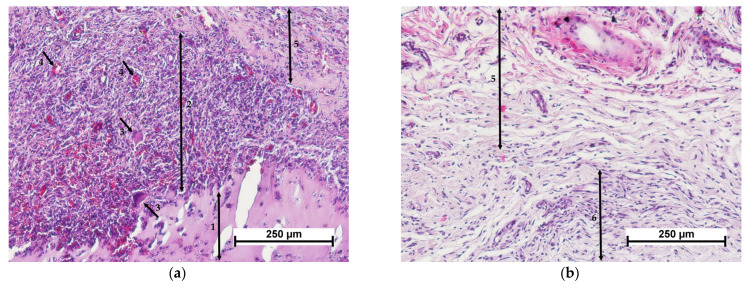

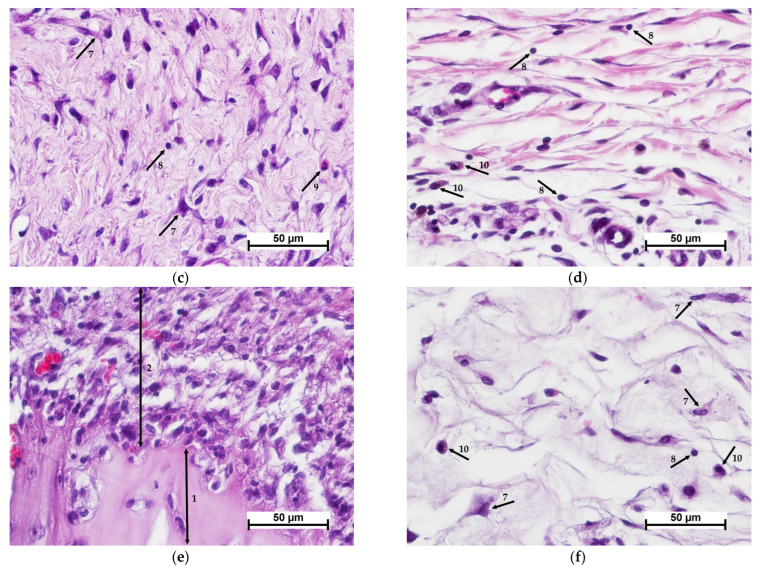
Histological picture—day 7. Comparison of cell-containing scaffold (**a**,**c**,**e**) and cell-containing collagen matrix (**b**,**d**,**f**). (**a**,**b**)—implant with surrounding tissue, general view, (**c**,**d**)—connective tissue structure surrounding the implant, (**e**)—cell-containing scaffold surrounded by resorbing cells, (**f**)—collagen matrix structure. Staining with hematoxylin–eosin. Note: (1)—scaffold; (2)—layer of resorbing cells (macrophages); (3)—giant multinucleated cells; (4)—blood vessels; (5)—connective tissue structure around the implant; (6)—collagen matrix; (7)—fibroblast; (8)—lymphocyte; (9)—granulocyte; (10)—macrophage.

**Figure 8:**
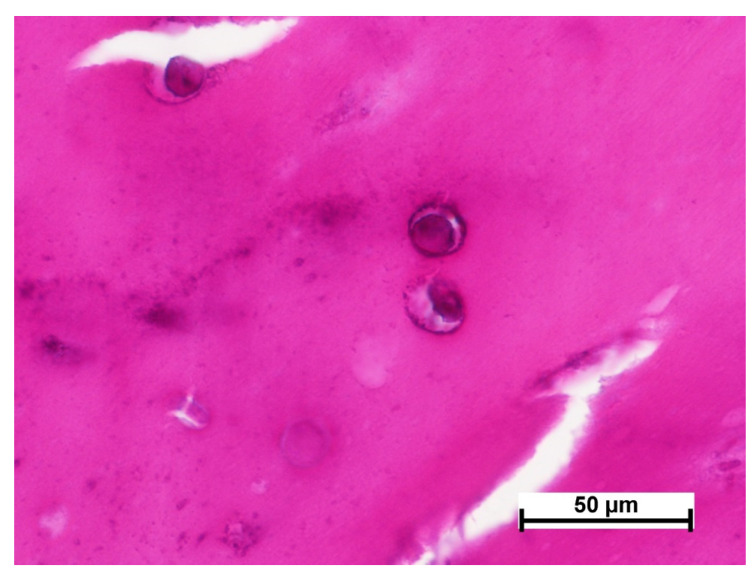
MSCs in cell scaffold 7 days after implantation. Staining with hematoxylin–eosin.

When studying the cellular experimental material—CCSs (scaffolds with encapsulated MSCs), it was found that, by 7 days after implantation, the CCS samples had become fragmented, and were surrounded by a dense layer of resorbing cells (macrophages and giant multinucleated foreign body cells) containing full blood vessels with small foci of hemorrhages between them (Figure 7a). The layer of resorbing cells was surrounded by newly formed connective tissue with active fibroblasts, slight lymphocytic infiltration and single granulocytes (Figure 7c). At the resorption border, the matrix was filled with cells showing signs of degenerative changes (Figure 7e). MSCs could be observed in the deep layers of the remaining matrix (Figure 8).

Comparison of the results of the cell-free material and CCS implantations revealed that the presence of MSCs in the structure of the experimental material had caused no evident effect on the implant resorption activity. In both cases, the material was partially resorbed and fragmented by day 7 of the experiment.

In contrast, the presence of MSCs in the control specimens (collagen matrix with MSCs) accelerated the biodegradation of the implanted material. After 7 days of the experiment, the border between the implant and the surrounding tissue was not clearly defined (Figure 7b). The surrounding connective tissue showed moderate edema, and weak lymphocytic infiltration with single granulocytes (Figure 7d). The peripheral part of the implant was replaced by newly formed connective tissue with active fibroblasts and foci of neovascularization. Active macrophages were detected in the matrix structure, while the predominant cells of fibroblastic origin were fibroblasts providing active synthesis of the intercellular matrix (Figure 7f). Thus, the processes of implant resorption and collagenogenesis were proceeding simultaneously, as in the cell-free control group, but more actively.

**Figure 9:**
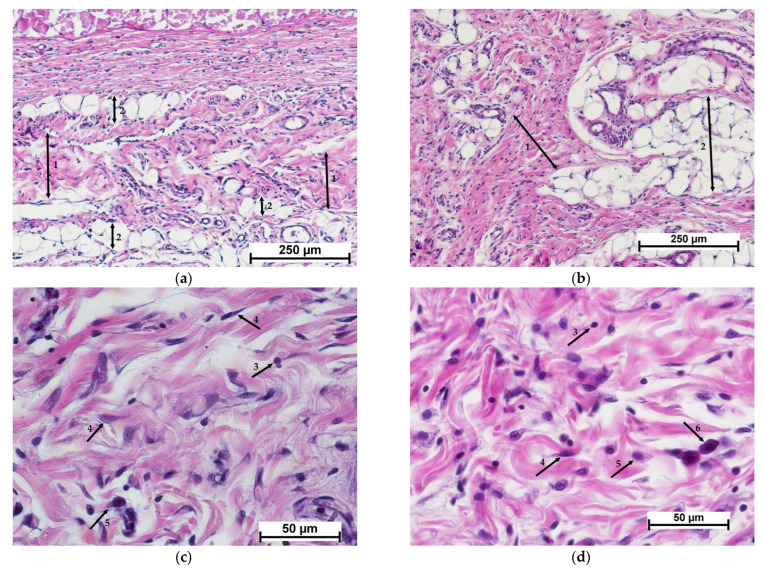
Histological picture—day 14. Comparison of cell-containing scaffold (**a**,**c**) and cell-containing collagen matrix (**b**,**d**). (**a**,**b**)—implant site, general view, (**c**,**d**)—connective tissue structure at the implantation site. Staining with hematoxylin–eosin. Note: (1)—connective tissue; (2)—adipose tissue; (3)—lymphocyte; (4)—fibroblast; (5)—macrophage; (6)—mast cell.

By day 14 after CCS implantation, as in the case of the cell-free material, connective tissue had completely replaced the matrix. The implant site showed dense irregular connective tissue with interlayers of adipose tissue (Figure 9a). This connective tissue showed mild edema, slight lymphocytic infiltration and single mast cells, together with collagen fibers of different maturity levels packed into bundles with fibroblasts and macrophages between them (Figure 9c). With MSCs present in the structure of the experimental material, connective tissue maturation was accelerated compared to the cell-free specimens. However, in these 14 day samples, the collagenogenesis process was still continuing, as evidenced by the presence of active fibroblasts [25,26].

When a control material with mesenchymal stem cells was implanted, implant resorption acceleration was also observed. Thus, by day 14, groups of adipocytes surrounded by dense irregular connective tissue were found at the site of the implanted material (Figure 9b). The connective tissue showed mild edema, collagen fibers of different maturity levels packed into bundles with fibroblasts and macrophages between them, slight lymphocytic infiltration, and the presence of single mast cells and granulocytes (Figure 9d).

**Figure 10:**
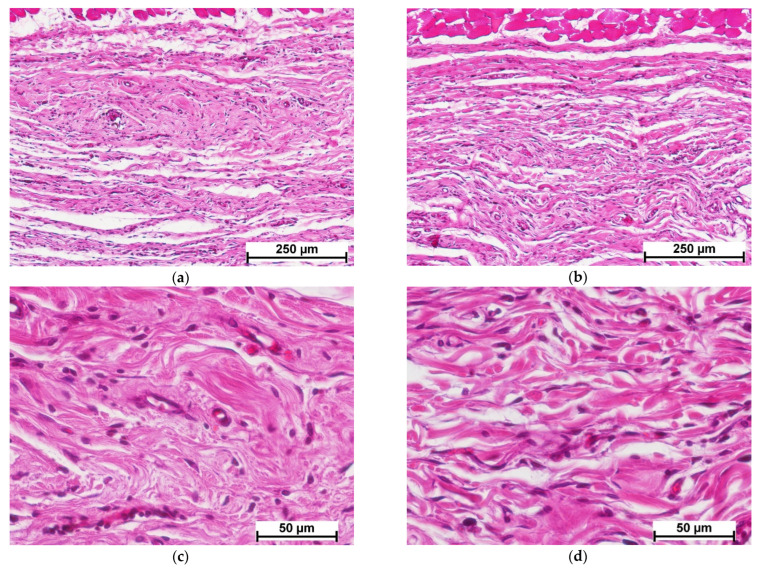
Histological picture—day 21. Comparison of cell-containing scaffold (**a**,**c**) and cell-containing collagen matrix (**b**,**d**). (**a**,**b**)—implant site, general view, (**c**,**d**)—connective tissue structure at the implant sites. Staining with hematoxylin–eosin.

On day 21 after CCS implantation, dense irregular connective tissue was detected at the implant site (Figure 10a). This connective tissue showed evident edema. The collagen fibers were characterized as mature, forming bundles with fibrocytes, single leukocytes, monocytes, granulocytes, and with mast cells between them (Figure 10a). A similar pattern was observed in the control specimens (Figure 10b,d). As a result, it may be concluded that, by study day 21, mature connective tissue had completely replaced the control and experimental materials.

Thus, morphological analyses showed that the subcutaneous implantation of control and experimental materials populated with mesenchymal stem cells had caused no acute inflammation over any of the observation periods. The inflammatory reaction was manifested as only weak or moderate lymphohistiocytic infiltration of the surrounding tissues. The presence of MSCs had accelerated control material biodegradation and accelerated connective tissue maturation in the CCS implantation area compared to the cell-free specimens.

### 3.4. Biotransformation of Cell-Containing Scaffolds

Based on the data obtained using transmission electron microscopy, the results were analyzed by calculating the proportion of the implant structure-forming portion (considering cells entering the field of view) to the total area of the analyzed image. This calculated “percentage of the implant structure-forming portion” reflects the density of the implant or of the tissues replacing it. It was found that the density of the studied specimens in the area of cell scaffold implantation dynamically increased over time (Table 1). At the same time, no density change could be observed at days 3 and 7 for the studied areas of specimens where the collagen matrix with MSCs had been implanted, this index increasing only by day 14. It was shown that the percentage of the implant structure-forming portion was markedly higher in the cell-containing scaffold samples than in the collagen matrix with MSCs at all analyzed periods. The difference in the processes of biodegradation and replacement by connective tissue as revealed by morphological analyses of the studied specimens may explain this discrepancy (see Section 3.3). There is no doubt that the changes occurring with the implants are related not only to their original structure, but primarily to the activity of cells: both the MSCs located in the implants and cells recruited from the surrounding tissues into the implant [27,28,29,30]. Our earlier results confirm the latter. MSCs were shown to change the scaffold structure and properties (including susceptibility to biodegradation) during long-term cultivation in vitro [4,31].

**Figure 11:**
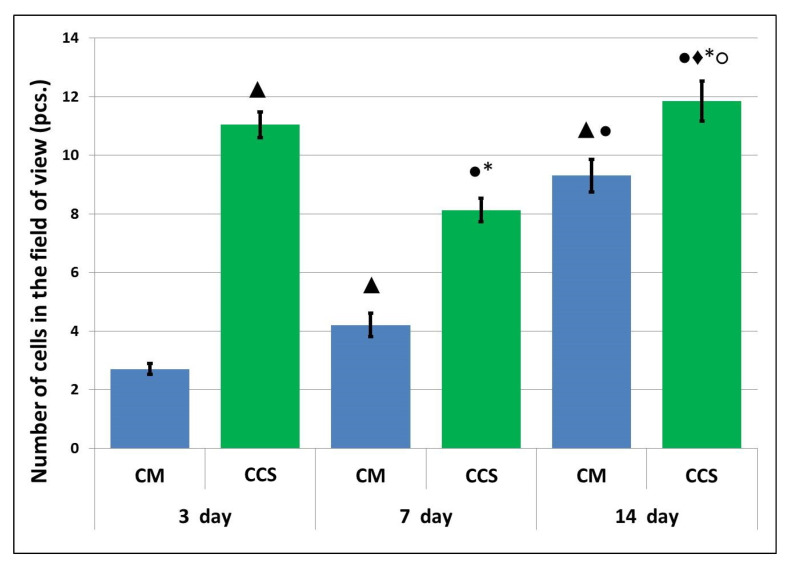
Number of cells per field of view (transmission microscopy). Note: CM—collagen matrix with MSC, CCS—cell-containing scaffold; ▲—*p* < 0.05 with 3-day CM; ●—*p* < 0.05 with 7-day CM; ♦—*p* < 0.05 with 14-day CM; *—*p* < 0.05—comparison with 3-day CCS; ○—comparison with 7-day CCS, Mann–Whitney U-test.

**Figure 12:**
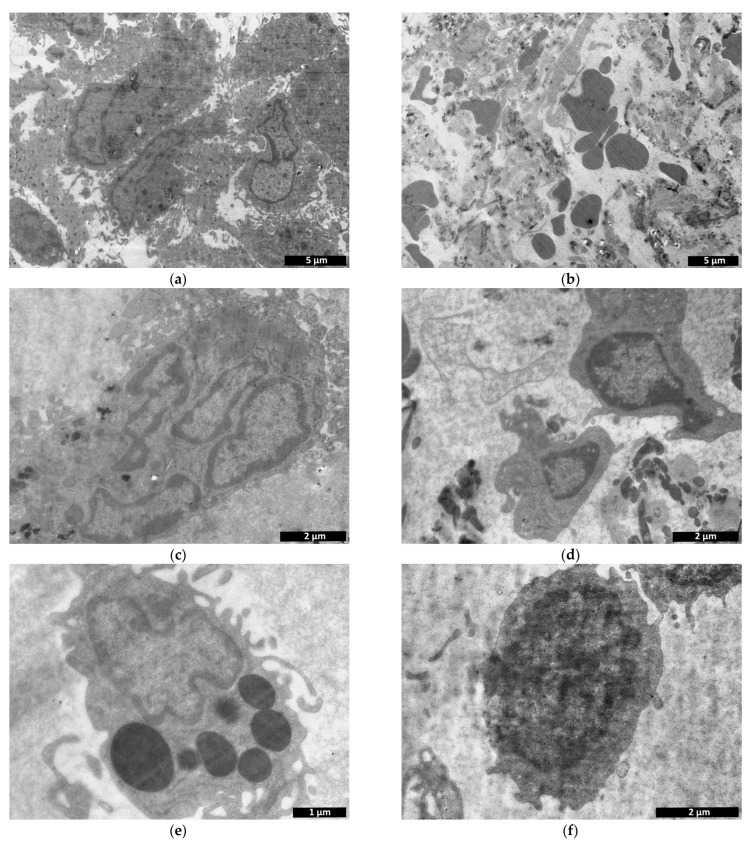
Comparison of experimental—cell-containing scaffolds (**a**,**c**,**e**) and controls—cell-containing collagen matrices (**b**,**d**,**f**): transmission microscopy, day 3. (**a**)—fibroblastic differon cells; (**b**)—erythrocytes in the intercellular space; (**c**) multinucleated cell; (**d**)—cells of fibroblastic origin; (**e**)—scaffold phagocytosing cell; (**f**)—lymphocyte.

Transmission electron microscopy revealed a large number of cells present within the implant structure on day 3 after implantation (Figure 11). Fibroblastic differon cells were among those detected (Figure 12a) along with large multinucleated cells (Figure 12c) and macrophages (Figure 12e), apparently phagocytosing the scaffold. A large number of fibroblastic cells evidenced the concurrent active synthesis of the intercellular matrix.

At the same time, the number of cells observed in the field of view for the control specimens was four times lower compared to the experimental specimens (Figure 11). Along with cells of fibroblastic origin (Figure 12d), a large number of erythrocytes (Figure 12b), single lymphocytes (Figure 12f) and macrophages were found. All this may evidence a reduced rate of matrix biodegradation compared to the CCS samples.

**Figure 13:**
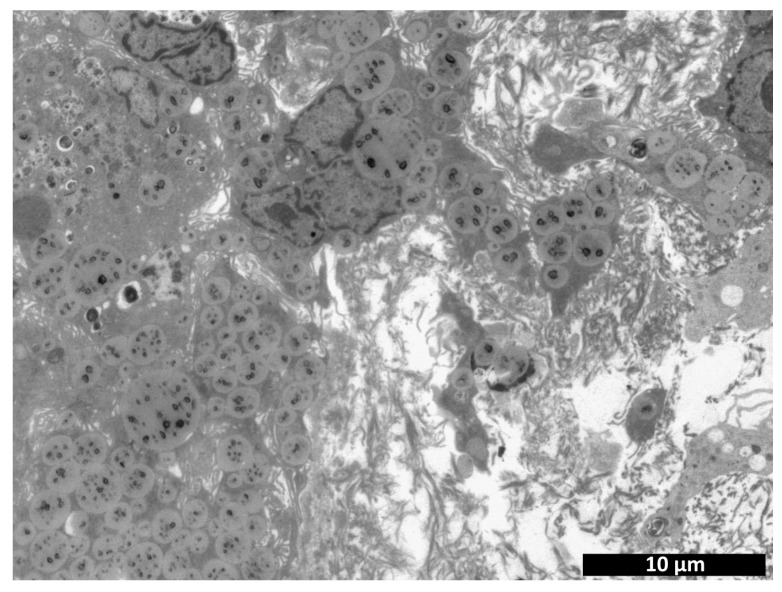
Vesiculation of scaffold (CCS), its removal by cells and replacement by collagen fibers 7 days after implantation.

**Figure 14:**
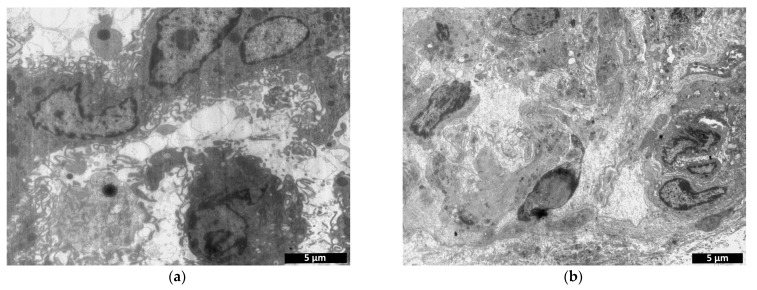
Comparison of experimental cells of fibroblastic origin—cell-containing scaffold (**a**) and control—cell-containing collagen matrix (**b**): transmission microscopy, day 7.

On day 7 after implantation, the experimental material retained its structure, and the number of cells observed per field of view had decreased by 26% compared to the previous period (Figure 11). The latter could be due to degradation and death of cells encapsulated in the scaffold. Despite this, vesiculation of the matrix was observed in one of the specimens, along with its removal by cells and replacement by collagen fibers (Figure 13). Fibroblastic differon cells were found in the matrix structure (Figure 14a) along with scaffold phagocytizing cells.

On day 7 after implantation of the control material, an increase in cell number was observed compared to day 3, but the number of cells in the control was still almost two times lower than in the experimental specimens (Figure 11). While no erythrocytes were detected in the material structure, cells of fibroblastic origin were observed (Figure 14b). The appearance of an amorphous substance and collagen fibers evidenced the resorption of the implant and its replacement by connective tissue.

**Figure 15:**
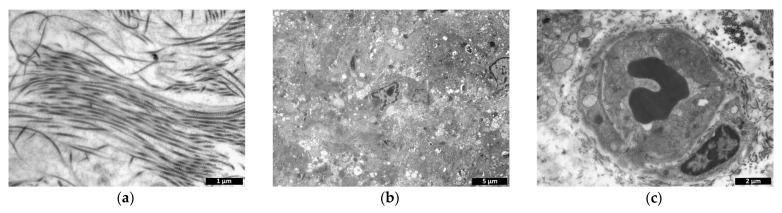
Cell-containing scaffold (transmission microscopy, day 14). (**a**)—collagen fibers, (**b**)—vesiculation and scaffold uptake by cells, (**c**)—capillary.

By study day 14, a large number of collagen fibers were detected at the experimental material implantation site (Figure 15a), and remnants of the material were found in phagocytic cells and in vesicles of the intercellular space (Figure 15b). The presence of capillaries evidenced neovascularization (Figure 15c).

**Figure 16:**
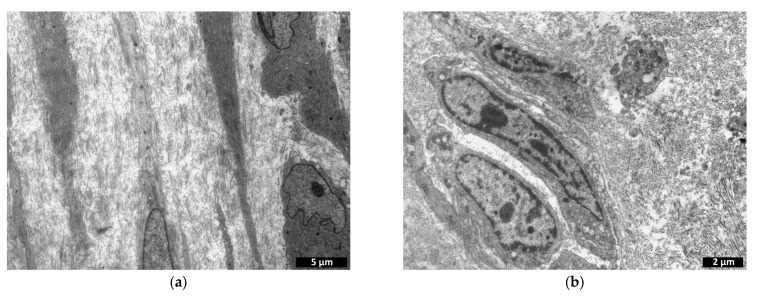

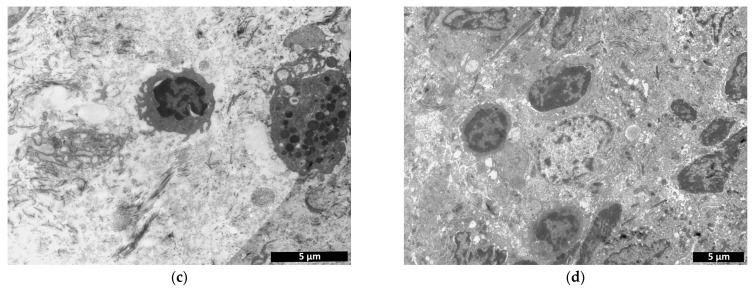
Comparison of experimental material—cell-containing scaffolds (**a**,**c**) and controls—cell-containing collagen matrices (**b**,**d**): transmission microscopy, day 14. (**a**,**b**)—cells of fibroblastic origin; (**c**)—lymphocyte and macrophage; (**d**)—lymphocytes and fibroblasts.

The number of cells had increased compared to the previous period and was comparable to the number observed on study day 3 (Figure 11). Fibroblastic differon cells (Figure 16a), lymphocytes, and macrophages (Figure 16c) were mainly found.

On day 14 after implantation, the numbers of cells in the control specimens had increased compared with those on days 3 and 7, but these numbers remained significantly lower than those of the experimental specimens for the same period (Figure 11). At the same time, as in the experimental specimens, matrix replacement by connective tissue components, cells and collagen fibers was observed at the implantation site (Figure 16c,d).

**Figure 17:**
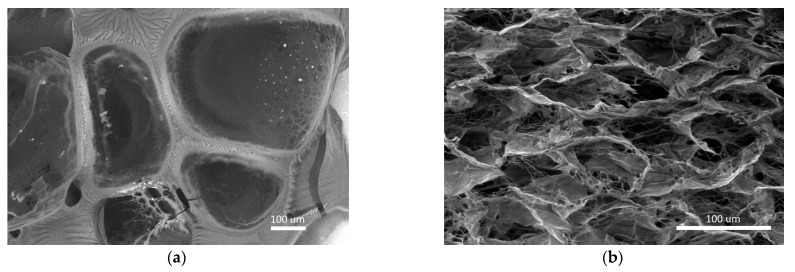
The porous structure of the studied materials (scanning electron microscopy): (**a**)—biopolymer scaffold, (**b**)—collagen matrices.

Summarizing the obtained results, we can say that cell recruitment to the material structure from the surrounding tissues was observed in both the control and experimental specimens. This evidences the integration of the materials into the tissues [32,33]. Based on the literature data, it can be argued that the presence of MSCs in the studied materials contributed to this. It is known that MSCs, implementing a paracrine function, secrete a number of biologically active substances providing for the recruitment of cells from surrounding tissues [34,35,36]. E.g., peptides secreted by MSCs promote the recruitment of circulating cells with angiogenic activity, leading to increased angiogenesis [37,38]. At the same time, the population of the studied and control specimens with recruited cells was different. Differences were observed in both the cell composition and cell numbers. These differences could be due to the different structural characteristics and compositions of the materials. Thus, the control material used in the study was represented by a porous biodegradable collagen matrix (“sponge”) consisting of horse tendon collagen, while the experimental scaffold-carrier was represented by a biodegradable biopolymer hydrogel with a three-dimensional structure formed on the basis of blood plasma cryoprecipitate and fish collagen (isolated from cod skins) (Figure 17). It should be noted that the ratio of fibrinogen, the main structure-forming protein, and collagen in the scaffold carrier composite was 22:1, respectively [4]. Thus, the materials used have certain differences of composition and properties. Literature data confirm that cell recruitment, including the composition and number of recruited cells, may vary depending on the composition and properties of the structural matrix/scaffold used [39,40]. The results of morphological analyses of the cell-free specimens of the control material and of the scaffold (3.2) also testify in favor of the latter statement. Thus, the scaffold was more actively populated with cells as early as day 7 compared to the collagen matrix.

We observed differences not only in cell recruitment, but also in the processes associated with the biodegradation of the studied materials and their transformation/remodeling after implantation into the tissues. Thus, it was shown that the processes of control material resorption and its replacement by connective tissue proceeded simultaneously. Under the same conditions of experimental specimen study, both resorption and collagenogenesis were evident. These differences may also be explained by the different compositions and properties of the studied materials. At the same time, the materials presented in the study may be considered to act as artificial extracellular matrices. It is known that cells within an extracellular matrix are involved in its reorganization. They are the source of enzymes breaking down the extracellular matrix components along with synthesizing and secreting building components, regenerating tissues and maintaining their homeostasis [41,42]. Therefore, it may be assumed that differences in the cellular occupancy of the material structure also caused differences in the resorption and remodeling processes. The data obtained using transmission microscopy support this statement. Thus, it was shown that the cells actively phagocytized the scaffold-carrying experimental material. At the same time, no similar phenomenon was observed in the control material. The relationship between the differences of material remodeling processes and cellular activity is also confirmed by the morphological analysis data. It was shown that on day 14 active fibroblasts prevailed in the cell-free scaffold group and foci of neovascularization were present, evidencing endothelial cell recruitment. In the group with the collagen matrix, fibrocytes and vascularization processes were observed for the same period. Comparative results of specimens and tissues in the area of cell-free collagen matrix implantation and of collagen matrix populated with MSCs, in turn, confirm the role of MSCs in the processes of matrix biodegradation and transformation. Thus, the biodegradation processes of the matrix populated with MSCs proceeded faster than those of the material without cells. Corresponding differences in these groups were observed for collagenogenesis, namely its acceleration in the presence of MSCs.

At the same time, despite the differences detected, the final effect at the end of the study was comparable between the groups, for the control (a clinically approved commercial product) and the experimental materials. This confirms the possibility for the future use of our experimental material (scaffold and scaffold carrier with MSCs) in clinical practice. This, along with the detected regenerative effect, provide grounds for further studies in order to determine the effectiveness of the presented experimental material for tissue regeneration.

## 4. Conclusions

The conducted morphological analyses using light-optical and ultrastructural methods showed that connective tissue components completely replaced the implants in both the experimental and control specimens. The replacement process was faster for the experimental implants. The presence of MSCs in the implant structure accelerated implant replacement by connective tissue in the control group, but had no evident effect on the biotransformation rate of the experimental material. The active formation of collagen fibers and absence of acute inflammation, taken together with the neovascularization, evidenced regeneration processes in the area of scaffold carrier or cell-containing scaffold implantation. The latter determines the prospects for the use of the presented tissue-engineered construct in regenerative medicine, in particular, for the purpose of soft tissue repair.

## Figures and Tables

**Table 1:** Dynamics of density changes after implantation.

Day	Percentage of the Implant Structure-Forming Portion to the Total Area of Analyzed Image	
	CM	CCS
day 3	69.86 ± 1.41	75.73 ± 2.01 ▲
day 7	69.52 ± 2.86	80.72 ± 2.37 ● *
day 14	83.46 ± 1.92 ▲ ●	88.85 ± 1.25 ♦ * ○

Note: CM—collagen matrix with MSCs, CCS—cell-containing scaffold; ▲—*p* < 0.05 with 3-day CM; ●—*p* < 0.05 with 7-day CM; ♦—*p* < 0.05 with 14-day CM; *—*p* < 0.05—comparison with 3-day CCS; ○—comparison with 7-day CCS, Mann–Whitney U-test.

## Data Availability

Not applicable.
